# Antibacterial and Antifungal Fabrication of Natural Lining Leather Using Bio-Synthesized Silver Nanoparticles from *Piper Betle* L. Leaf Extract

**DOI:** 10.3390/polym15122634

**Published:** 2023-06-09

**Authors:** Ngoc-Thang Nguyen, Tien-Hieu Vu, Van-Huan Bui

**Affiliations:** 1 Department of Textile Material and Chemical Processing, School of Textile-Leather and Fashion, Hanoi University of Science and Technology, 1 Dai Co Viet, Hanoi 11615, Vietnam; huan.buivan@hust.edu.vn (V.-H.B.); 2 Department of Leather and Footwear Technology, Ho Chi Minh City Industry and Trade College, 20 Tang Nhon Phu, Ho Chi Minh 71210, Vietnam; vutienhieu@hitu.edu.vn (T.-H.V.)

**Keywords:** silver nanoparticles, green synthesis, *Piper betle* L. leaf, pig lining leather, antibacterial activity, antifungal activity

## Abstract

Leather is often used to make comfortable shoes due to its soft and breathable nature. However, its innate ability to retain moisture, oxygen and nutrients renders it a suitable medium for the adsorption, growth, and survival of potentially pathogenic microorganisms. Consequently, the intimate contact between the foot skin and the leather lining surface in shoes, which are subject to prolonged periods of sweating, may result in the transmission of pathogenic microorganisms and cause discomfort for the wearer. To address such issues, we modified pig leather with silver nanoparticles (AgPBL) that were bio-synthesized from *Piper betle* L. leaf extract as an antimicrobial agent via the padding method. The evidence of AgPBL embedded into the leather matrix, leather surface morphology and element profile of AgPBL-modified leather samples (pLeAg) was investigated using colorimetry, SEM, EDX, AAS and FTIR analyses. The colorimetric data confirmed that the pLeAg samples changed to a more brown color with higher wet pickup and AgPBL concentration, owing to the higher quantity of AgPBL uptake onto the leather surfaces. The antibacterial and antifungal activities of the pLeAg samples were both qualitatively and quantitatively evaluated using AATCC TM90, AATCC TM30 and ISO 16187:2013 test methods, approving a good synergistic antimicrobial efficiency of the modified leather against *Escherichia coli* and *Staphylococcus aureus* bacteria, a yeast *Candida albicans* and a mold *Aspergillus niger*. Additionally, the antimicrobial treatments of pig leather did not negatively impact its physico-mechanical properties, including tear strength, abrasion resistance, flex resistance, water vapour permeability and absorption, water absorption and desorption. These findings affirmed that the AgPBL-modified leather met all the requirements of upper lining according to the standard ISO 20882:2007 for making hygienic shoes.

## 1. Introduction

Leather is a natural material obtained through the tanning process of a hide of an animal, bird or reptile. Leather has been extensively employed for making various items, such as footwear, clothing, bags, wallets and other accessories. Owing to its softness, breathability and high moisture-absorbing properties, leather provides comfort to the wearer [1,2,3,4]. However, due to its good moisture absorption, sweat-containing proteins may serve as a nutrient source that supports the growth of bacteria and fungi on leather goods, particularly within shoes where the foot skin is in close contact with the lining surface [5,6,7,8]. In addition, the collagen fiber network within the leather structure provides suitable conditions of moisture, temperature and oxygen for microorganism growth. Moreover, leather footwear products are commonly not washed during use, leading to the accumulation and proliferation of microorganisms, resulting in unpleasant odors, discoloration, reduced mechanical strength and skin diseases in wearers [9,10,11]. The hot and humid climate in Vietnam provides proper conditions for bacterial and fungal growth on leather goods during storage, transportation and use. Hence, the antimicrobial characteristics of leather footwear products are concerns among both consumers and enterprises.

To overcome such issues, antimicrobial finishing of leather footwear products using various antimicrobial agents and treatment methods is often employed [2,3,8,9,10,11]. Many antibacterial and antifungal agents have been investigated for their effectiveness in leather treatment, including silver nanoparticles, zinc oxide nanoparticles, polymer compounds containing quaternary ammonium, chitosan and its derivatives [12,13,14,15,16]. These agents work through contact mechanisms and cell membrane disruption of microorganisms [17,18,19]. Although some chemical antimicrobial agents are used in the tanning process, their main function is to prevent the biodegradation of the leather rather than providing antimicrobial properties [2,12]. Furthermore, the use of some antibacterial and antifungal agents has been limited due to health and environmental concerns [20,21]. Therefore, developing high-performance antimicrobial agents that are effective against broad-spectrum bacterial and mold strains and environmentally friendly for leather material is imperative [20,21,22].

Thus, the appropriate selection of antimicrobial agents and treatment methods is important for creating durable antimicrobial leather materials that effectively prevent undesirable microbial growth while minimizing negative impacts on the material properties and the environment. To address these concerns, bio-synthesized silver nanoparticles (AgNPs) treated on leather have attracted significant attention from scientists due to their broad antimicrobial activity and durability to microorganisms [17,21,23,24]. The synthesis of green AgNPs involves the utilization of bio-reductants derived from natural resources such as plants, algae and microorganisms. Incorporating AgNPs into the collagen fiber matrix of leather enhances the material’s long-lasting antimicrobial effects and exhibits low toxicity towards mammalian cells, making them suitable for producing high-quality leather goods [2,3].

Recently, we reported on a green approach to fabricating silver nanoparticles using *Piper betle* L. leaf extract (PBL) as bio-reductants to reduce Ag+ ions into silver metal, which adheres fully to the principles of green chemistry [25]. The spherical shape and narrow size distribution of the obtained silver nanoparticles (AgPBL) showed good synergistic antibacterial activity against three common bacterial strains, including *Escherichia coli*, *Pseudomonas aeruginosa* and *Staphylococcus aureus*. In this work, we further evaluated the antifungal activity of AgPBL against one mold strain (*Aspergillus niger*) and one yeast strain (*Candida albicans*). We then investigated a simple approach to apply AgPBL onto tanned pig leather utilized for shoe lining (Le) by a padding method. The padding method was selected to apply antimicrobial treatment to the pig leather because it is suitable for use during the wet finishing stage of leather production. The presence and distribution of AgPBL on the pig leather surface were evaluated using various analytical techniques, namely colorimetry, scanning electron microscopy (SEM), energy-dispersive X-ray spectroscopy (EDX), atomic absorption spectroscopy (AAS) and Fourier-transform infrared spectroscopy (FTIR). The antibacterial and antifungal efficacy of the modified leather was assessed qualitatively and quantitatively using established protocols for antimicrobial testing of textile and leather materials in accordance with AATCC TM90, AATCC TM30 and ISO 16187:2013 against two bacterial strains (*Escherichia coli* and *Staphylococcus aureus*) and two fungal strains (*Aspergillus niger* and *Candida albicans*). To the best of our knowledge, there is no report available on the antibacterial and antifungal treatment of pig leather using bio-synthesized AgPBL for shoe lining application.

## 2. Materials and Methods

### 2.1. Materials

Analytical grade silver nitrate (Ag(NO)_3_ 99.99%, Aladdin Biochemical Technology Co., Ltd., Shanghai, China) and *Piper betle* L. leaves (PBL, Hai Duong Province, Vietnam) were used for the preparation of the silver nanoparticles (AgPBL) under optimal conditions according to our published research [25]. The samples of pristine pig leather (Le) in the wet-blue tanning form were obtained from Hung Thai Brothers Tannery Co., Ltd., Ho Chi Minh, Vietnam. The leather was then split in our laboratory using a DS818-420L leather splitter (Wenzhou Dashun Machinery Manufacture Co., Ltd., Zhejiang, China) to obtain a uniform thickness of 1 ± 0.1 mm, which is proper for use as shoe lining material. The pig lining leather was further cut into small pieces (100 × 100 mm) and dried in a Mesdan M250-RH conditioning chamber (Brescia, Italy) at 65% RH and 25 °C for 24 h before being stored in a plastic bag for further study. In all experiments, double distilled water from an EYELA Still Ace SA-2100E (Tokyo Rikakikai Co., Ltd., Tokyo, Japan) was employed as the solvent. The dedicated medium (SCDLP), Luria-Bertani (LB) agar and Sabouraud dextrose agar (SDA) were supplied by Oxoid (Thermo Fisher Scientific Inc., Waltham, MA, USA). All microbial strains, including two bacterial strains *Escherichia coli* (*E. coli*, ATCC 25922) and *Staphylococcus aureus* (*S. aureus*, ATCC 29213, ATCC, Manassas, VA, USA), a mold strain *Aspergillus niger* (*A. niger*, ATCC 16404) and a yeast strain *Candida albicans* (*C. albicans*, ATCC 10231) were provided from the School of Biotechnology—International University, NTT Hi-Tech Institute—Nguyen Tat Thanh University, Institute of Tropical Biology—Vietnamese Academy of Science and Technology.

### 2.2. Synthesis and Application of AgPBL to Pig Lining Leather

We have previously described the bio-synthesis of the silver nanoparticles using *Piper betle* L. leaf extract as bio-reductants [25]. Briefly, dried PBL leaves were boiled with double distilled water at a ratio of 1:40 for 15 min. The mixture was then filtered through Whatman No. 1 filter paper. Subsequently, the resulting PBL filtrate underwent centrifugation at 10,000 rpm for 20 min to eliminate any insoluble residues. Prior to the nanosilver synthesis, the PBL supernatant was further diluted 20 times with double distilled water. For the AgPBL synthesis, 1 mL of 10 mM AgNO_3_ solution was reduced with 10 mL of the diluted PBL extract and allowed to stand for 4 h at room temperature in the dark to avoid any unnecessary photochemical reactions. The mixtures containing silver nanoparticles were purified by centrifugation at 16,000 rpm, 5 °C for 30 min, followed by washing with double distilled water in a UT-106H Ultrasonic Cleaner (Sharp Corporation, Osaka, Japan). The purification process was repeated twice to remove residual reagents, and the AgPBL was collected and re-dispersed in double distilled water to achieve various concentrations for further investigation.

**Figure 1:**
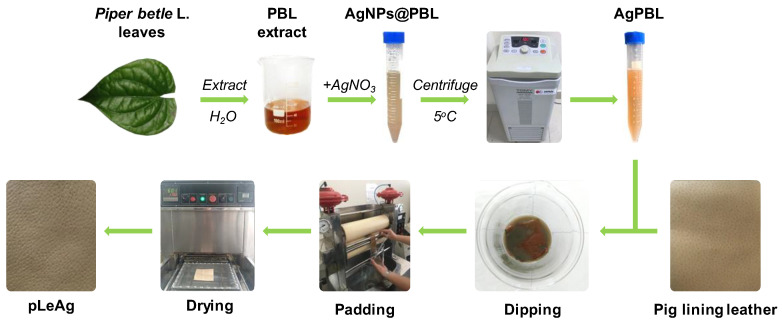
Schematic illustration of the processes of AgPBL synthesis and its application onto pig lining leather, namely pLeAg.

In the next step, the pig lining leather samples were treated with the bio-synthesized AgPBL solutions using the padding method. The leather samples were dipped in AgPBL solutions with various concentrations (160, 80, 40 and 20 μg/mL) for 30 min at a liquor-to-leather ratio of 5:1 (*w*/*w*). The wet pickups were set at 70%, 80% and 90%, and the padded samples were then dried at 105 ± 3 °C for 3 min using SDL mini-drier 398 laboratory thermo-fixation (SDL Atlas China, Shenzhen, China). The dipping–padding–drying processes of the leather samples were repeated two times. All processed leather samples were conditioned at 65% RH and 25 °C in a Mesdan M250-RH (Mesdan SpA, Brescia, Italy) conditioning chamber for 24 h before storage in plastic bags for microbiological analysis. The processes of synthesizing AgPBL and applying it onto pig lining leather are depicted in Figure 1.

### 2.3. Analytical Methods

#### 2.3.1. Characterization of the AgPBL

The UV-vis absorption spectrum of the AgPBL was acquired employing a UV-1800 spectrophotometer (Shimadzu, Kyoto, Japan) with a quartz cuvette in the range of 300–700 nm and with a resolution of 1 nm.

The diameters of AgPBL nanoparticles were recorded by transmission electron microscopy (JEOL JEM-1400, JEOL, Tokyo, Japan). A suspension of AgPBL in double distilled water was sonicated for 2 min and dropped onto a Cu-grid for TEM analysis.

#### 2.3.2. Characterization of the Modified Pig Leather

The morphology and chemical content of the control and AgPBL-modified leather samples after platinum sputtering were inspected using SM-6510LV JEOL (JEOL, Tokyo, Japan) scanning electron microscope (SEM) coupled to Oxford EDS Microanalysis System (Oxford Instruments NanoAnalysis, High Wycombe, UK).

A PinAAcle 900T atomic absorption spectrometer (AAS, PerkinElmer, Waltham, MA, USA) was employed to record silver content in the AgPBL-modified leather sample.

A Nicolet 6700 spectrometer (Thermo Scientific, Waltham, MA, USA) was utilized to record the FTIR spectra of the control and modified leather samples within the 4000–500 cm^−1^ range.

Ci4200 spectrophotometer (X-rite, Grandville, MI, USA) was employed to report both colorimetric data (L*, a* and b*) and color differences (ΔE*) of the leather samples before and after treatment with the AgPBL solutions. In the CIELab color space, L* is lightness from brightest white (100) to darkest black (0); a* is the color ratio from red (+) to green (−) and b* is the color ratio from yellow (+) to blue (−). The total color difference was determined using Equation (1):

(1) $$∆E=\sqrt{{∆L}^{*2}+{∆a}^{*2}+{∆b}^{*2}}$$

where ΔL*, Δa* and Δb* represent the colorimetric differences in L*, a* and b* values, respectively, of the blank and modified leather samples.

#### 2.3.3. Physico-Mechanical Characterization

The AgPBL-modified leather was evaluated for its physico-mechanical properties, which are essential requirements for shoe lining materials, in accordance with the standard ISO 20882:2007 [26]. These properties include tear strength (ISO 17696), abrasion resistance (ISO 17704), flex resistance (ISO 17694), lining water vapour permeability and absorption (ISO 17699), and lining water absorption and desorption (ISO 22649). The physico-mechanical tests were carried out at the Institute of Footwear Research, Vietnam.

### 2.4. Antibacterial and Antifungal Activities

#### 2.4.1. Bio-Synthesized Silver Nanoparticles (AgPBL)

The antifungal activities of the AgPBL against yeast *C. albicans* and mold *A. niger* were studied by well diffusion method and disk diffusion method, respectively, following the Clinical Laboratory Standard Institute guidelines [27]. For the anti-yeast test, a suspension of *C. albicans* strain (0.1 mL, 10^6^ CFU/mL) was spread uniformly on plates containing Sabouraud dextrose agar (SDA). Next, five 6 mm diameter holes were made using a sterile cork borer. Then, 60 μL of AgPBL solutions at various concentrations (100, 50 and 25 μg/mL), a standard antibiotic (Streptomycin, 80 μg/mL) as positive control and double distilled water as negative control were poured into their respective wells. The zone of inhibition (ZOI) produced by *C. albicans* was recorded after 24 h of incubation at 37 °C.

For the anti-mold test of *A. niger*, the disk diffusion method was performed using sterile 6 mm paper discs loaded with AgPBL solutions (100, 50 and 25 μg/mL) and double distilled water as the negative control. Aliquots of 0.1 mL of *A. niger* strain (approximately 10^6^ CFU/mL) were spread on SDA agar plates, followed by the placement of the prepared paper discs on their surface. The zone of inhibition against A. niger was measured after 7 and 14 days of growth at 28 °C. The results were expressed as the mean ± standard deviation (SD) of three independent tests.

The zone of inhibition (ZOI) was calculated based on Equation (2):

W = (T − D)/2 (2)

where

W is the width of clear zone of inhibition, mm;

T is the total diameter of the test specimen and clear zone, mm;

D is the diameter of the test specimen, mm.

#### 2.4.2. The Modified Pig Leather

The antibacterial and antifungal activities of the control and AgPBL-modified leather samples against *E. coli*, *S. aureus*, *C. albicans* and *A. niger* were investigated qualitatively and quantitatively using established protocols for testing the antimicrobial activity of textile and leather materials, including AATCC TM90, AATCC TM30 and ISO 16187:2013 test methods [28,29,30].

For qualitative tests (AATCC TM90 and AATCC TM30), the disk diffusion method was used to determine the zone of inhibition. A volume of 0.1 mL of each organism strain (approximately 10^6^ CFU/mL) was spread on Luria-Bertani (LB) agar plates for bacteria and SDA agar plates for fungi. Next, the control and AgPBL-modified leather samples were positioned on the surface of agar plates, which were subsequently incubated at 37 °C for 24 h for bacteria and *C. albicans*, and at 28 °C for 7 and 14 days for *A. niger*. Zones of inhibition around and on the leather samples were visually examined.

For quantitative tests (ISO 16187:2013), the static challenge protocol was performed to determine the percentage reduction of bacteria. Six control samples (pristine leather, Le) and six modified leather samples (pLeAg) at each AgPBL concentration were prepared with dimensions of 25 × 25 × 1 mm and placed in individual sterile glass flasks. To each flask, 1 mL of bacterial suspension with a concentration of 5.0 × 10^5^ CFU/mL was added. At the initial time (zero contact time), three control samples and three modified leather samples were collected and washed out with 20 mL of dedicated medium (SCDLP). The remaining six flasks were incubated for 24 h at 37 °C (24 h contact time) and then washed out with 20 mL of the SCDLP medium. All flasks were tightly capped and shaken in an incubator shaker at 120 rpm and 37 °C for 30 s. A series of ten-fold dilutions of the bacterial sample solutions were made using NaCl 0.85% aqueous solution, and 100 μL of each diluted bacterial solution was spread over LB agar plates. After incubating the plates at 37 °C for 24 h, the surviving bacteria were enumerated by counting their colonies. The bacterial reduction percentage was measured using Equation (3):

R = (C_t_ − T_t_) × 100%/C_t_ (3)

where

R is the bacterial reduction percentage, %;

C_t_ and T_t_ are the average number of colonies of three control samples and three test samples after 24 h, respectively, CFU/mL.

## 3. Results and Discussion

### 3.1. Synthesis and Antimicrobial Activity of AgPBL

**Figure 2:**
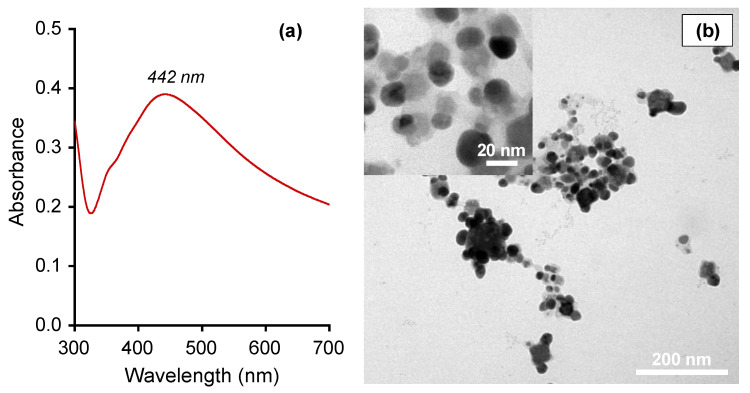
(**a**) UV-vis spectrum and (**b**) TEM micrographs of AgPBL with different magnification ×30 k and ×200 k (inset).

In previous research, we optimized the conditions for the bio-synthesis of silver nanoparticles from PBL leaf extract and evaluated their antibacterial activities against three bacterial strains, including *E. coli*, *P. aeruginosa* and *S. aureus* [25]. In the current work, we re-fabricated AgPBL under the optimized conditions and characterized its properties using UV-vis, TEM and antifungal activity analyses. The UV-Vis spectrum in Figure 2a indicated a maximum absorption peak at 442 nm due to the surface plasmon resonance (SPR) of spherical silver nanoparticles [21,22,25]. The TEM image in Figure 2b confirmed well-dispersed, spherical-shaped nanoparticles with a fairly uniform size of about 20 nm. The well-dispersion of AgPBL was attributed to the bio-constituents in the PBL extract, which effectively prevented nanoparticle agglomeration and stabilized them.

**Figure 3:**
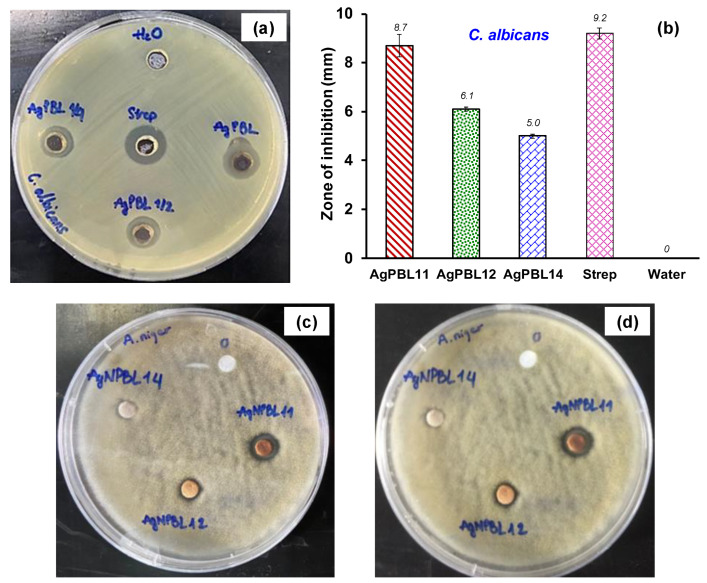
Antifungal activities of the AgPBL: (**a**) Well diffusion method displaying the anti-*C. albicans* action of AgPBL11 (AgPBL 100 μg/mL), AgPBL12 (AgPBL 50 μg/mL), AgPBL14 (AgPBL 25 μg/mL), Streptomycin (80 μg/mL, positive control) and H_2_O (negative control); (**b**) Mean zone of inhibition of AgPBL against *C. albicans* (±SD, *n* = 3); Disk diffusion method displaying the anti-*A. niger* action of AgPBL after (**c**) 7 days and (**d**) 14 days of incubation.

To assess the antifungal activity of the AgPBL, one mold strain (*A. niger*) and one yeast strain (*C. albicans*) were exposed to various AgPBL concentrations (100, 50 and 25 μg/mL) via the plate diffusion method. As shown in Figure 3a–d, the double distilled water (a negative control) exhibited no antifungal activity in terms of inhibition zone, while the AgPBL and the Streptomycin (Strep) obviously revealed ZOI. It was evident that AgPBL showed promising antifungal activity against both tested fungi. A comparison of the ZOI size for each fungal strain indicated a tendency for decreased antifungal action with decreasing AgPBL concentration, although this tendency was not proportional to the change in AgPBL concentration. For instance, reducing the AgPBL concentration by 50% and 75% resulted in a ZOI reduction for *C. albicans* of 29.9% and 42.5%, respectively. The results observed from Figure 3c,d after 7 and 14 days of the antifungal tests against *A. niger* revealed clearly that mold spores could not grow on the AgPBL-impregnated paper disks at any concentration. However, the paper disk impregnated with 25 μg/mL AgPBL solution did not exhibit a clear inhibition zone, indicating that the antifungal activity of AgPBL against *C. albicans* was better than against *A. niger*. The antifungal properties exhibited by bio-synthesized AgNPs were consistent with the findings of previous studies [31,32].

### 3.2. Coloration and Characteristics of the AgPBL-Modified Pig Leather

The presence of AgPBL on pig lining leather was visually observed through the color change of the sample during the treatment of the leather with bio-synthesized AgPBL solutions using the padding method. Indeed, the color of the leather was changed significantly from bright yellow to light brownish color. To illustrate the effect of the wet pickup and AgPBL concentration on the coloration of the leather surface, the colorimetric data (L*, a*, b*) and color differences (ΔE*) of the leather samples were evaluated, as shown in Table 1. The color of the blank leather was bright yellow with relatively high L*, a* and b* values of 62.42, 9.77 and 23.86, respectively. To compare with the blank leather, the AgPBL-modified leather samples showed smaller L*, a* and b* values, indicating a browning effect of the AgPBL on the leather samples. The ΔE* of the modified samples increased with higher wet pickup and AgPBL concentration, owing to the higher quantity of AgPBL uptake onto the leather surfaces.

**Figure 4:**
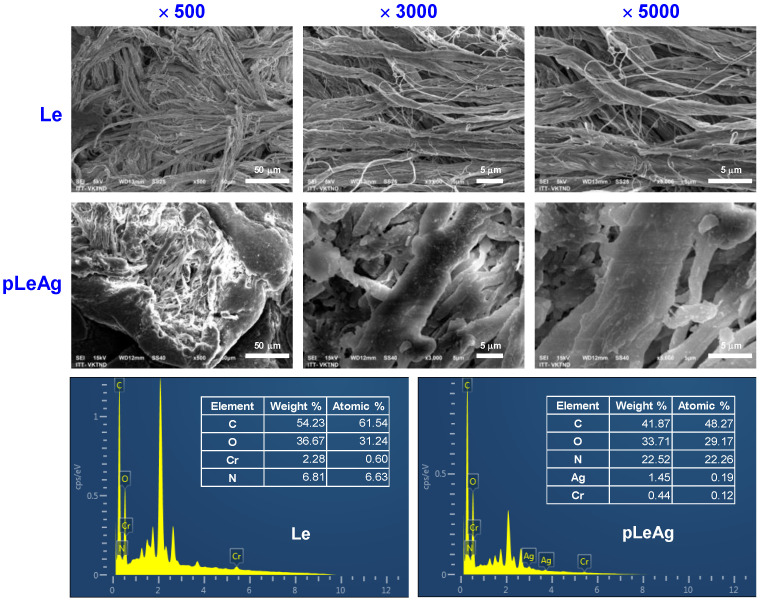
The SEM micrographs at different magnifications of ×500, ×3000 and ×5000, and EDX spectra of the blank leather (Le) and the AgPBL-modified leather sample (pLeAg).

To get visual evidence of the AgPBL embedded onto the leather, leather surface morphologies and element profile of the blank and modified leather samples were investigated. As shown in Figure 4, the blank and modified leather revealed a distinctive hierarchically suprafibrillar structure of the collagen fiber strands. The micrographs of modified leather samples (Figure 4) revealed the occurrence of nanoparticles loosely attached to the leather surface. In dark-field SEM images, nano metals usually appear as bright spots due to their strong light scattering [22,33]. However, the silver nanoparticles employed in this work were about 20 nm in size and could impregnate deeply into the collagen fiber strands of the leather matrix. As a result, they could be challenging to detect in SEM images, which only provide information on the surface morphology of the sample. Therefore, EDX analytical technique was performed to validate the presence of nanosilver on the leather sample after treatment.

The EDX spectrum of the blank leather in Figure 4 revealed no Ag signal, but the occurrence of a Cr signal confirming that the pig leather was chrome-tanned leather. In contrast, the EDX spectra of the AgPBL-modified leather sample via the padding treatment (pLeAg) clearly showed a strong signal of elemental silver at 3 keV which authenticated the existence of AgPBL on the leather surface [9,12]. The EDX spectra of those modified samples confirmed again that the bright points in the SEM images were AgPBL.

To evaluate the amount of AgPBL adhered to the pig leather after padding treatment, the AAS analysis was performed, and the result was presented in Table 2. The data indicates that the total silver content in the padded sample was around 380 mg/kg, whereas the blank leather sample did not contain any silver.

**Figure 5:**
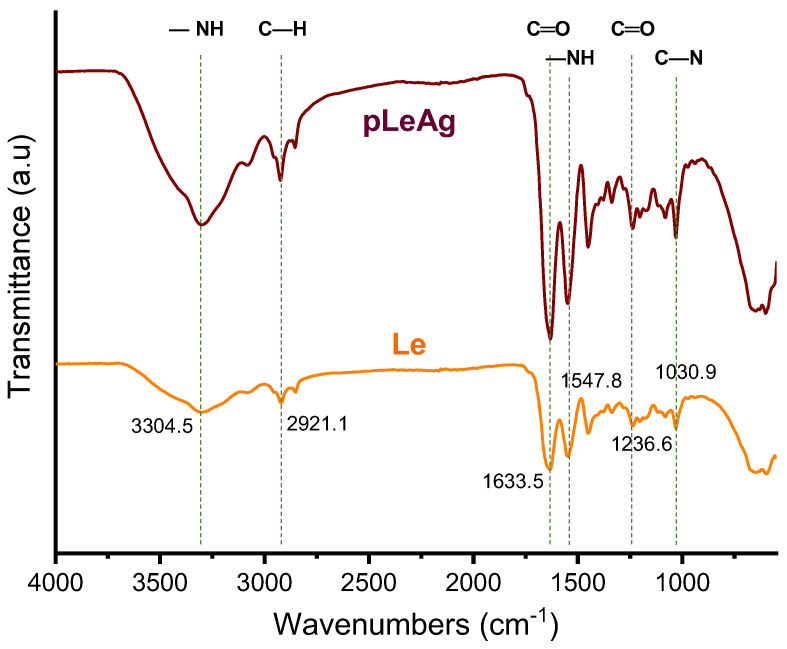
The FTIR spectra of the Le and pLeAg samples.

In order to determine the possible interaction between AgPBL and the functional groups of collagen proteins on the modified leather, the FTIR analyses of Le and pLeAg samples were carried out, and the spectra were given in Figure 5. The characteristic peaks corresponding to the functional groups of the collagen proteins in the Le sample at 3304.5, 2921.1, 2852.3, 1633.5, 1547.8, 1236.6 and 1030.9 cm^−1^ were assigned to –NH, –CH_3_, =CH_2_, –C=O, –NH, –C=O (in amide III) and C–N (in amine) groups, respectively [10,13,34]. Compared to the blank leather, the pLeAg spectrum exhibited similarity in their characteristic peaks, except for the stronger peak intensities, suggesting the chemical structure of the leather was mostly unchanged. The collagen proteins of leather contain polar groups on the side chains of their constituent amino acid residues, and as such, the amide and carboxylate functional groups of these residues have a tendency to bind with metal atoms [10,12]. Thus, AgPBL could be absorbed onto the leather surface through the electrostatic interaction of Ag^+^ ions with the negative charge of RCOO^−^ or lone-pair electrons of N atoms of amino acids. In addition, the formation of hydrogen bonding between the amide and carboxylate groups of the collagen proteins with the appropriate functional groups of the organic layer existing on the AgPBL surface also contributes to these binding interactions. The shifted peaks of the functional groups in the modified leather samples could be attributed to the interaction of heavy silver atoms with the amino and amide groups of collagen protein molecules, resulting in an increase in peak intensity [12]. The results of coloration, SEM, EDX, AAS and FTIR measurements for both blank and modified leather samples were consistent with each other, providing strong evidence for the incorporation of AgPBL into the leather matrix.

### 3.3. Antibacterial and Antifungal Efficacy of the AgPBL-Modified Pig Leather

Leather is a naturally hydrophilic material that offers a potential medium for the growth of microorganisms such as bacteria, yeasts and molds. The utilization of silver nanoparticles on the lining of leather could avoid the risk of infection and extend the lifetime of leather products by inhibiting microorganism growth. In this research, we investigated the antibacterial and antifungal efficacy of AgPBL-modified leather against *E. coli*, *S. aureus*, *C. albicans* and *A. niger*. Both qualitative (the disk diffusion method) and quantitative (the static challenge protocol of dynamic contact method) tests were employed to determine the antimicrobial activities of the modified leather.

#### 3.3.1. Antibacterial Efficacy

**Figure 6:**
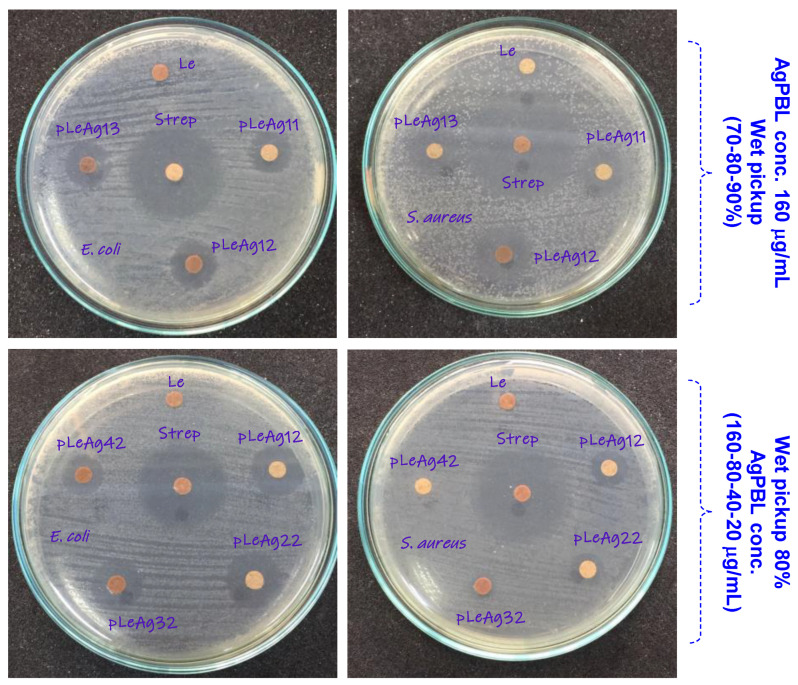
The photographs showing zone of inhibition of the Le, pLeAg and Streptomycin (80 μg/mL) treated leather samples against *E. coli* and *S. aureus*, with change in the wet pickup and AgPBL concentration.

The AgPBL-modified leather samples obtained via the padding method were evaluated for their antibacterial activities against a gram-negative bacterium *E. coli* and a gram-positive bacterium *S. aureus*. The effect of the wet pickup and AgPBL concentration on the antibacterial efficacy of the modified leather was examined, and results were shown in Figure 6 and Table 3.

A quick survey across Figure 6 evidences that all the AgPBL-modified leather samples and the Strep-treated leather exhibited obviously ZOI against *E. coli* and *S. aureus*, whereas the pristine leather (Le) showed no activity at all. These findings demonstrate the antibacterial efficacy of the AgPBL-modified leather in this work. As shown in Figure 6, the ZOI of the modified leather samples did not change significantly with varying wet pickups. The pLeAg12 sample with a wet pickup of 80% exhibited the highest ZOI of 8.7 ± 0.21 and 10.2 ± 0.24 mm against *E. coli* and *S. aureus*, respectively. Accordingly, the wet pickup of 80% was selected for further evaluation of the antibacterial activity.

Table 3 presents the results of the antibacterial tests of the padded leather samples, which showed that higher AgPBL concentrations led to larger inhibition zones. However, the relationship between AgPBL concentration and inhibition zone was not proportional. Indeed, as compared to the pLeAg12 sample, when the AgPBL concentrations were decreased by 50% and 75%, the ZOI of pLeAg22 and pLeAg32 samples against *E. coli* decreased by 0.9% and 42.0%, respectively. Additionally, there was a slight difference in the inhibition zone between leather samples treated with AgPBL concentrations of 160 and 80 μg/mL. Moreover, the antibacterial results in terms of inhibition zone clearly demonstrated that the AgPBL-modified leather samples exhibited higher effectiveness against gram-negative bacteria than gram-positive bacteria, which is in line with previous findings regarding the bactericidal properties of bio-synthesized silver nanoparticles [25,35].

**Figure 7:**
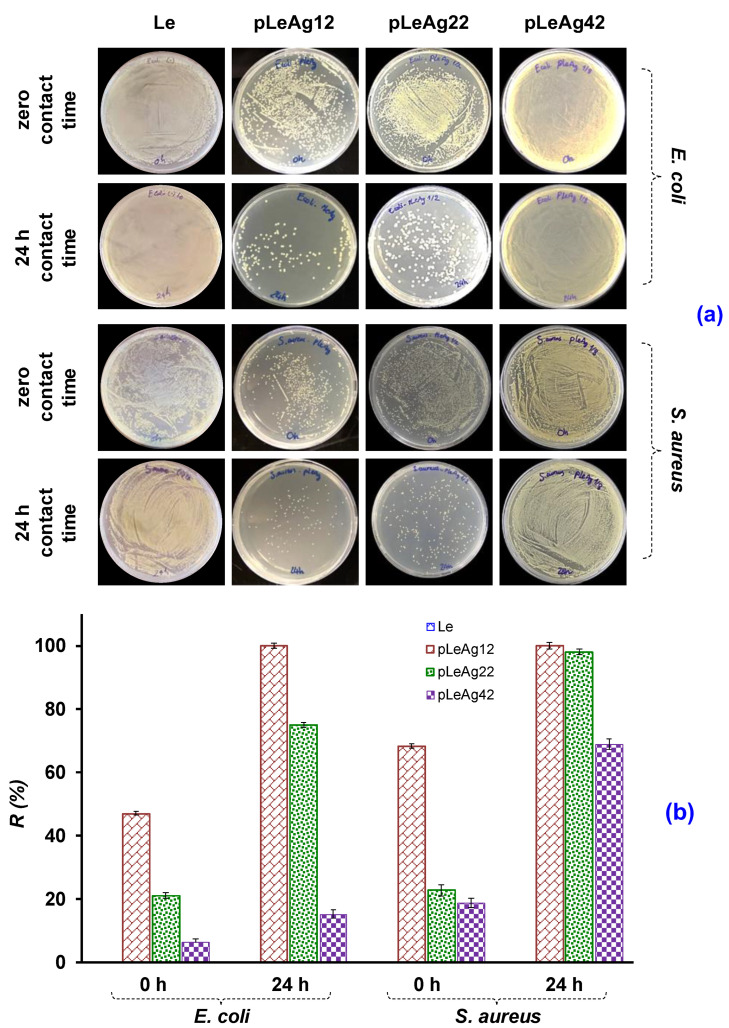
(**a**) The photographs of bacterial growth in the nutrient agar plates and (**b**) The bacterial reduction percentage (%R) of the Le and AgPBL-modified leather samples against *E. coli* and *S. aureus*.

The leather samples treated with various AgPBL concentrations at the wet pickup of 80% were subjected to the quantitative test to determine the bacterial reduction percentage. This test was performed at 0 h and 24 h contact times between the sample and the test bacteria inoculate. As shown in Figure 7a, the Petri dishes for 24 h contact time revealed that both tested bacteria grew well on the Petri dishes of blank leather, indicating that Le did not possess bacterial inhibition. Based on the agar’s turbidity, the pLeAg12 samples showed the highest antibacterial activity, while the pLeAg42 samples exhibited the lowest.

By counting the number of colonies of tested bacteria on agar dishes, the bacterial reduction percentage of the treated leather samples with different concentrations of AgPBL was plotted in Figure 7b. The results showed that the pLeAg12 samples treated with 160 μg/mL AgPBL exhibited the highest antibacterial efficiency against both *E. coli* and *S. aureus* after 0 h contact time, with bacterial reduction percentages of 47.02% and 68.43%, respectively. Moreover, both achieved a 100% antibacterial rate after a 24 h contact time.

The antibacterial activity of pLeAg22 samples treated with 80 μg/mL AgPBL decreased significantly against *E. coli* after both 0 h and 24 h contact time. In comparison to pLeAg12 samples, the bactericidal rates of pLeAg22 samples against *E. coli* and *S. aureus* decreased by 24.8% and 1.88%, respectively, after 24 h contact time. Similarly, pLeAg42 samples treated with 20 μg/mL AgPBL exhibited a sharp decrease in antibacterial activity against both tested bacteria. Compared to the pLeAg12 samples, the bactericidal rates of the pLeAg42 samples against *E. coli* and *S. aureus* after 24 h contact time declined by 84.93% and 31.13%, respectively. Despite the lack of a zone of inhibition in the AgPBL-modified leather sample at a low concentration (20 μg/mL AgPBL) observed by the qualitative antibacterial method, the sample still possessed a weak antibacterial ability.

Based on the obtained antibacterial results, the quantitative antibacterial efficiency of the modified leather samples is strongly influenced by the initial AgPBL concentration in the treated solutions. The antibacterial efficiency also depends on the tested bacteria and is not proportional to the change in AgPBL concentration. The findings are consistent with the qualitative antibacterial evaluation of the modified leather samples. The suitable concentration of AgPBL for pig lining leather treatment was 160 μg/mL to achieve 100% antibacterial efficiency after 24 h contact time.

#### 3.3.2. Antifungal Efficacy

**Figure 8:**
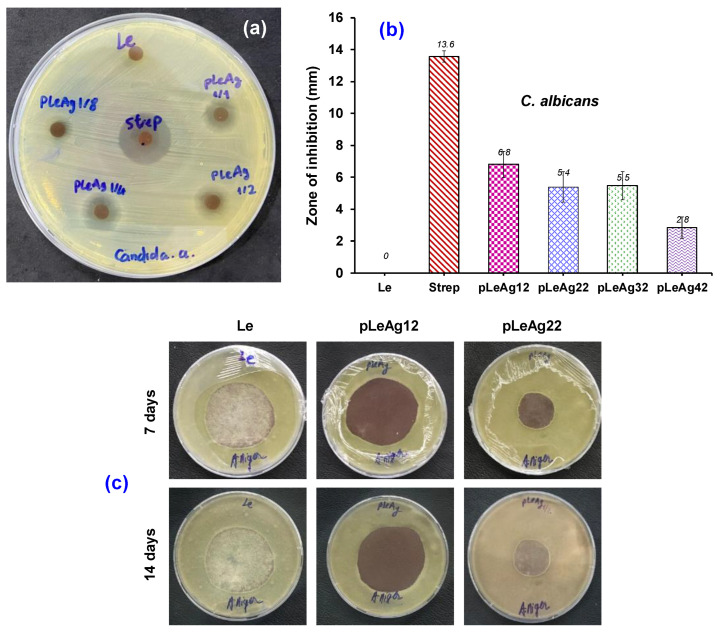
(**a**) Antifungal activities of the Le, pLeAg and Strep-treated leather samples against *C. albicans* with change in the AgPBL concentration; (**b**) Mean zone of inhibition against *C. albicans* (±SD, *n* = 3); (**c**) Antifungal activities of the Le, pLeAg12 and pLeAg22 samples against *A. niger* after 7 and 14 days of incubation.

The AgPBL-modified leather samples were evaluated for their antifungal activities against one yeast strain (*C. albicans*) and one mold strain (*A. niger*) according to AATCC TM30. Figure 8 depicted that the blank leather revealed no antifungal activity, whereas both AgPBL-modified leather samples and the Strep-treated leather exhibited considerable antifungal efficiency against the tested fungi. Figure 8a,b confirmed that a decrease in the treated AgPBL concentration resulted in a reduction of antifungal efficacy against the *C. albicans* strain, as evidenced by a decrease in the ZOI value by 20.6% and 19.6% when the treated AgPBL concentration was reduced by 50% and 75%, respectively. As shown in Figure 8c, the antifungal tests against *A. niger* after 7 and 14 days revealed that the pLeAg12 sample padded with 160 μg/mL AgPBL solution did not show any mold spore germination or growth. However, the pLeAg22 sample padded with 80 μg/mL AgPBL solution exhibited mold spore growth on its surface after 7 days, which was obviously seen after 14 days of incubation. These findings confirm once again the strong dependence of the antimicrobial effectiveness of the AgPBL-modified samples on the AgPBL concentration in the antimicrobial-treated solution. Consequently, pig leather treated with 160 μg/mL solution at a wet pickup of 80% was selected for further evaluation of its physico-mechanical properties.

### 3.4. The Physico-Mechanical Properties of the AgPBL-Modified Pig Leather

The blank leather and AgPBL-modified leather samples were subjected to various tests following standard specifications to evaluate their physico-mechanical properties. The experimental data were compared with the requirements of the shoe lining material according to the standard ISO 20882:2007. As presented in Table 4, the mechanical properties of the AgPBL-modified leather including tear strength, abrasion resistance and flex resistance were nearly identical to those of pristine leather. This is because the antimicrobial treatment process used in this research did not alter the chemical structure of pig leather, and nanosilver adheres to the leather matrix solely through physical bonds. The mechanical data of the modified samples met all the standard requirements for shoe lining, and the tear strength was even over 200% of the standard requirement.

Similarly, the antimicrobial treatment of leather with AgPBL did not affect its water absorption and water desorption. The water absorption of the leather samples before and after treatment was around 90% of the standard requirements for the lining. However, this lining layer is typically combined with a porous layer in the shoe upper structure, resulting in an enhancement of its water absorption capacity. The water desorption of the leather samples was excellent, reaching over 160% of the standard requirement. The water vapour permeability and water vapour absorption of the leather samples were very good, exceeding 150% of the standard requirements for upper lining material. Pore expansion on the modified leather surface after the padding process led to an increase in its water vapour permeability. The leather material swells when it absorbs water and then shrinks during the drying process. However, the collagen fiber strands’ shrinkage is greater than that of the pores, leading to pore expansion.

In general, the antimicrobial treatments of pig leather did not negatively impact its physico-mechanical properties. The AgPBL-modified leather met all the requirements according to the standard ISO 20882:2007 for making shoe linings. The padding method is suitable for tanneries, which is carried out at the wet finishing stage (dyeing, oiling, etc.) in a rotary drum, then padded and transferred to the drying finishing stage in a drying chamber.

## 4. Conclusions

This research provided a simple approach for the fabrication of highly effective antimicrobial pig leather modified with bio-synthesized silver nanoparticles as an antimicrobial agent via the dipping–padding–drying processes. The effect of the wet pickup and AgPBL concentration on the coloration, antimicrobial activity and physico-mechanical properties of the modified leather were investigated according to standard methods. The experiment data validated that the higher wet pickup and AgPBL concentration led to a browner color and enhanced antimicrobial efficacy of the AgPBL-modified leather against both bacteria (*E. coli* and *S. aureus*) and fungi (a yeast *C. albicans* and a mold *A. niger*). These observed results could be attributed to the increased uptake of AgPBL into the leather matrix, which was verified through SEM, EDX, AAS, and FTIR analyses. However, the antimicrobial treatment of pig leather using the padding method did not have any adverse effect on its physico-mechanical properties, and it met the ISO 20882:2007 standard’s requirements for the upper lining. Therefore, based on the efficient antimicrobial and suitable physico-mechanical properties, the AgPBL-modified pig leather meets the criteria for making upper lining in hygienic shoe production.

## Figures and Tables

**Table 1:** Colorimetric data, color differences and images of the AgPBL-modified leather samples in comparison with the blank pig leather.

Sample	Wet Pickup (%)	AgPBL (μg/mL)	L*	a*	b*	ΔE*	Real Images
Le	-	-	62.42	9.77	23.86	0	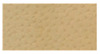
pLeAg11	70	160	57.94	9.13	21.38	2.33	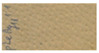
pLeAg12	80		57.97	9.75	21.12	2.41	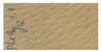
pLeAg13	90		57.01	9.27	21.38	2.66	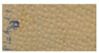
pLeAg12	80	160	57.97	9.75	21.12	2.41	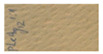
pLeAg22		80	59.85	9.29	22.27	1.39	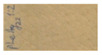
pLeAg32		40	59.94	9.79	23.07	1.15	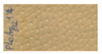
pLeAg42		20	60.01	9.54	23.74	1.04	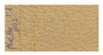

**Table 2:** Total silver content of the leather samples.

Sample	Wet Pickup (%)	AgPBL (μg/mL)	Total Silver Content (mg/kg)
Le	-	-	0
pLeAg12	80	160	379.0 ± 4.6

**Table 3:** The ZOI of AgPBL-modified leather samples against *E. coli* and *S. aureus* (±SD, *n* = 3).

Sample	Wet Pickup (%)	AgPBL (µg/mL)	*E. coli*	*S. aureus*
Vis	-	-	0	0
Strep	-	-	21.10 ± 1.10	29.00 ± 0.73
pLeAg11	70	160	8.40 ± 0.37	9.60 ± 0.33
pLeAg12	80		8.70 ± 0.21	10.20 ± 0.24
pLeAg13	90		8.20 ± 0.22	9.10 ± 0.36
Strep’	-	-	23.30 ± 0.92	24.00 ± 1.02
pLeAg12	80	160	11.40 ± 1.10	8.40 ± 0.33
pLeAg22		80	11.20 ± 0.24	7.30 ± 0.22
pLeAg32		40	10.50 ± 1.00	2.10 ± 0.08
pLeAg42		20	8.40 ± 0.37	-

**Table 4:** The physico-mechanical properties of the pristine leather and AgPBL-modified pig leather.

No	Properties	Unit	Le	pLeAg	ISO 20882:2007 Requirements
1	**Tear strength** (ISO 17696)	N	32	32.5	lining ≥ 15 N
	*Compare to the pristine leather (Le)*	%	100.0	101.6	
	*Compare to ISO 20882:2007*	%	213.3	216.7	
2	**Abrasion resistance** (ISO 17704)	cycles	Without hole through the thickness of the material component		25,600 cycles dry 12,800 cycles wet
3	**Flex resistance** (ISO 17694)	cycles	15,000 cycles dry without visible damage		Dry 15,000 cycles without visible damage
4	**Lining water vapour permeability** (ISO 17699)	mg/cm^2^.h	3.13	3.62	WVP of lining ≥ 2.0 mg/cm^2^.h
	*Compare to the pristine leather (Le)*	%	100.0	115.7	
	*Compare to ISO 20882:2007*	%	156.0	181.0	
5	**Lining water vapour absorption** (ISO 17699)	mg/cm^2^	21.0	20.8	WVA of lining ≥ 8.0 mg/cm^2^
	*Compare to the pristine leather (Le)*	%	100.0	99.0	
	*Compare to ISO 20882:2007*	%	263.0	260.0	
6	**Lining water absorption** (ISO 22649)	mg/cm^2^	54.1	53.9	absorption ≥ 70 mg/cm^2^
	*Compare to the pristine leather (Le)*	%	100.0	99.6	
	*Compare to ISO 20882:2007*	%	90.1	89.3	
7	**Lining water desorption** (ISO 22649)	%	97.1	97.2	desorption ≥ 60 %
	*Compare to the pristine leather (Le)*	%	100.0	100.1	
	*Compare to ISO 20882:2007*	%	161.8	162	

## Data Availability

The data presented in this study are available on request from the corresponding author.
